# Supplementary material for: Let me Google that for you: a time series analysis of seasonality in internet search trends for terms related to foot and ankle pain
Source: J Foot Ankle Res. 2015 Jul 3;8:27. doi: 10.1186/s13047-015-0074-9 (PMC4490673; doi:10.1186/s13047-015-0074-9)
Supplement: Additional file 1: — Search term data and analysis codes. [file 13047_2015_74_MOESM1_ESM.zip]

Research article

Let me Google that for you: a time series analysis of seasonality in internet search trends for terms related to foot and ankle pain

Scott Telfer[1]*, James Woodburn[2]

1. Research Fellow, Institute of Applied Health Research, Glasgow Caledonian University, Glasgow, UK
2. Professor of Rehabilitation, Institute of Applied Health Research, Glasgow Caledonian University, Glasgow, UK

*Corresponding author

Institute for Applied Health Research, Glasgow Caledonian University, Cowcaddens Road, Glasgow, UK, G4 0BA

Tel: +44(0)141 331 8475

Email: scott.telfer@gcu.ac.uk; scott.telfer@gmail.com

Word count: 2655

Abstract

Background: The analysis of internet search traffic may present the opportunity to gain insights into general trends and patterns in information seeking behaviour related to medical conditions at a population level. For prevalent and widespread problems such as foot and ankle pain, this information has the potential to improve our understanding of seasonality and trends within these conditions and their treatments, and may act as a useful proxy for their true incidence/prevalence characteristics. This study aimed to explore seasonal effects, general trends and relative popularity of internet search terms related to foot and ankle pain over the past decade.

Methods: We used the Google Trends tool to obtain relative search engine traffic for terms relating to foot and ankle pain and common treatments from Google search and affiliated pages for major northern and southern hemisphere English speaking nations. Analysis of overall trends and seasonality including summer/winter differences was carried out on these terms.

Results: Searches relating to general foot pain were on average 3.4 times more common than those relating to ankle pain, and twice as common as searches relating to heel pain. Distinct seasonal effects were seen in the northern hemisphere, with large increases in search volumes in the summer months compared to winter for foot (p=0.004, 95% CI[22.2-32.1]), ankle (p=0.0078, 95% CI[20.9-35.5]), and heel pain (p=0.004, 95% CI[29.1-45.6]). These seasonal effects were reflected by data from Australia, with the exception of ankle pain. Annual seasonal effects for treatment options were limited to terms related to foot surgery and ankle orthoses (p=0.031, 95% CI[3.5-20.9]; p=0.004, 95% CI[7.6-25.2] respectively), again increasing in the summer months.

Conclusions: A number of general trends and annual seasonal effects were found in time series internet search data for terms relating to foot and ankle pain. This data may provide insights into these conditions at population levels.

Keywords: Foot pain; ankle pain; Google Trends; plantar faciitis; ankle sprain; insoles; foot orthotics

Introduction

Foot and ankle pain that results from trauma, musculoskeletal disease or a neurological condition is highly prevalent and a significant burden at both the personal and healthcare provider level [1]. The role of the foot as the primary load bearing structure and the ankle as the most distal large joint in the lower limb kinetic chain means that pain and subsequent functional disability at these sites can have a dramatic effect on an individual's mobility, activity levels, and therefore independence [2]. In chronic conditions such as arthritis, foot and ankle pain resulting from disease activity can be hugely debilitating and lead to a significant reduction in quality of life [3].

Understanding seasonal and long term changes in foot and ankle pain incidence is of considerable importance, and identifying these patterns may aid in the planning and provision of care. For example, it is a common belief that cold and damp winter weather makes joints stiffer and more painful [4], and increased activity levels in the summer may lead to a higher incidence of overuse injuries [5, 6].

Over the past two decades the growth of the internet has made information relating to health and medicine more widely accessible [7]. Individuals now regularly search online for medical information, and this can be for a number of reasons, including: a desire for reassurance, to develop a greater understanding, or to obtain a second opinion [8]. Prior consultation with a medical professional before carrying out these types of searches is particularly low in people under 36 years of age [8]. This type of search traffic data is potentially a rich source of information for healthcare researchers looking to analyse trends relating to different medical conditions [9]. The utility of this data has only just begun to be explored; however notable results have been reported, for example, in using the data to attempt to detect outbreaks of influenza [10].

To facilitate access to this type of data, Google has provided a tool called Google Trends (http://www.google.com/trends/). This tool allows access to internet search patterns by analysing a portion of all web queries on the Google search website and its affiliated sites. The relative volume of searches using particular terms over time can be obtained and downloaded for further analysis. A recent systematic review of the literature reported that 70 healthcare related studies using Google Trends or its forerunners have been published since the tool's launch in 2008, with interest increasing rapidly [9].

Our aim in this study was to explore this internet search data for insights into trends in search volume for terms related to foot and ankle pain, selected specific conditions, and common treatments over the past 10 years. We hypothesized *a priori* that there may be seasonal components to these time series data, including differences between winter and summer search volumes, and explored overall trends.

Methods

This study did not directly involve human participants therefore specific ethical approval was not required. The checklist provided in Nuti et al. [9] was used as a basis for search strategy reporting. We used the Google Trends web interface to obtain search traffic data for a number of terms relating to foot and ankle pain and its treatment based on lay search terms. Our search terms were limited to English language only, and searches were carried out on 2014/12/15. All query categories were used. A full list of the search strings used has been included in Table 1. To capture data relating to general foot and ankle pain we used three strings: FOOT PAIN, ANKLE PAIN and HEEL PAIN. We included PLANTAR FACIITIS and ANKLE SPRAIN terms as examples of common, pain causing foot and ankle conditions for reference, but it should be noted that this was not intended to be a comprehensive analysis of internet searches relating to these conditions or any other potential condition that could lead to lower extremity

pain. Such an analysis is beyond the scope of the current study and in our opinion would be better explored at condition specific level with similarly specific hypotheses developed *a priori*. We also included search terms that related to common treatment options for foot pain, these were: FOOT ORTHOTICS, INSOLES, and FOOT SURGERY; and for ankle pain: ANKLE ORTHOSES, ANKLE EXERCISES, ANKLE STRETCHES, and ANKLE SURGERY.

For each search term, weekly search volumes from 2004/01/10 to 2014/12/15 were downloaded in .csv format for the United Kingdom, United States of America, Canada and Australia as the major English speaking nations in the northern and southern hemispheres. It should be noted that Google trends provides only relative data, not the absolute number of searches for each term. To assess the relative volume of search terms in the same group (Table 1), combined worldwide datasets were also downloaded.

Data analysis

Data processing and statistical analysis was carried out using R (V3.1.1) [11]. All figures were produced using the ggplot2 package [12]. To allow full reproducibility of these results, the original .csv data files and analysis scripts have been included as Supplementary Materials.

To obtain a single time series for the northern hemisphere, weighting factors based on population and mean internet penetration for the time period 2004-2012 [13] were applied (see supplementary materials for further details). Data from individual countries were included from the time point where consistent data, defined as an absence of large variations to and from zero in the time series, were being collected. At least 3 years of consistent, weekly data were required for a time series to be included and taken forward for analysis.

Seasonal decomposition of each search term time series was initially carried out by local regression (LOESS) to allow visual inspection of the data components. The Mann-Kendall trend test was use to detect overall trends significantly larger than the variance in the data for each search term (α=0.05). To determine if there were significant seasonal components, an exponential smoothing state space model with Box-Cox transformation, autoregressive-moving-average errors, trend and seasonal components (TBATS) was fitted to the data. Periodograms were produced to identify key seasonal cycles. In addition, two-way Wilcoxon signed-rank tests were applied to determine if there were significant differences between search volumes in the summer (June-September) and winter (November-February) months (α=0.05). Winter/summer months were reversed for Australian data.

Results

Results for all time series are summarised in Table 2.

Pain

Overall time series curves for the search terms in the pain group normalised to relative worldwide search volumes are shown in Figure 1, and seasonal time series decomposition with periodograms in Figure 2. On average, searches for FOOT PAIN were 3.4 times more common than those for ANKLE PAIN, and 2 times more common than those for HEEL PAIN. Overall increasing trends and an annual seasonal effect were detected for FOOT PAIN searches, and in the northern hemisphere these increased by 35% in the summer months (p=0.004, 95% CI[22.2-32.1]). A similar increase of 21% in summer was seen in the Australian data, however this did not reach statistical significance (p=0.25, 88% CI[-3.6-41.9]), likely due to the limited number of data points available in this series (2010 onwards). For ANKLE PAIN, an overall increasing trend, annual seasonal effects and an increase of 31% in the summer months

(p=0.0078, 95% CI[20.9-35.5]) were found in the northern hemisphere only, although again the Australian time series data was limited to ~4 years. In the case of HEEL PAIN, overall increasing trends, annual seasonal effects were seen for both time series, and in the northern hemisphere an increase in searches of 58% was seen for the summer months. Summer/winter differences did not reach significance in the Australian data (p=0.25, 75% CI[15.9-21.4]), although there was an overall increase in the summer median of 25%.

Conditions

Overall time series curves for search terms in the condition group normalised to worldwide search volumes are shown in Figure 3, and seasonal time series decomposition with periodograms in Figure 4. For PLANTAR FACIITIS, significant increasing trends were seen in both northern hemisphere (p<0.001) and Australian time series (p<0.001), as were seasonal effects. A significant (p=0.004, 95% CI[21.5-37.4]) increase in the summer months of 30% were seen in the northern hemisphere data, along with a non-significant (p=0.063, 95% CI[5.2 - 13.6]) increase of 8% in the Australian data. Searches for ANKLE SPRAIN terms were ~20 times more common than those for PLANTAR FACIITIS. Significant increasing trends for ANKLE SPRAIN terms were seen (p<0.001 northern hemisphere, p=0.027 Australia) as were seasonal components. There was a significant (p=0.004, 95% CI[14.5-33.7]) summer increase in search volume of 33% in the northern hemisphere, however this was not reflected in the Australian time series, where a non-significant summer decrease of 9% was found (p=0.125, 88% CI[7.6-18.7]).

Foot treatment

Overall time series curves for search terms in the treatment group normalised to worldwide search volumes are shown in Figure 5 and seasonal time series decomposition with periodograms in Figure 6. Searches for INSOLE terms over the time period were, on average, 13.7 and 6.8 times more popular than FOOT ORTHOTIC and FOOT SURGERY respectively. A significant (p=0.001) negative trend was seen in the

FOOT ORTHOTIC northern hemisphere time series, suggesting a decrease in the relative popularity of this term (there was insufficient data in the Australian time series for analysis). In contrast, a large increasing trend ($p<0.001$) in the popularity of INSOLE terms were seen in both the northern hemisphere and Australian data series (both $p<0.001$), with no seasonal components detected. No trends were seen in the northern hemisphere data for FOOT SURGERY, although an annual seasonal component was detected, with a significant ($p=0.031$, 95% CI[3.5-20.9]) increase of 18% in the summer months. There was insufficient data in the Australian time series for analysis.

Ankle treatment

Overall time series curves for search terms in the treatment group normalised to worldwide search volumes are shown in Figure 7 and seasonal time series decomposition with periodograms in Figure 8. There was insufficient data available to carry out any analysis for ANKLE STRETCH terms, as well as insufficient data in the Australian time series for the remainder of the ankle treatment search terms. There was a significant increasing trend in the ANKLE ORTHOSES search term ($p<0.001$) in the northern hemisphere along with an annual seasonal effect with an average increase of 8% in the summer months ($p=0.004$, 95% CI[7.6-25.2]). No significant trend was detected in the time series for ANKLE EXERCISE terms (p=0.067), and although a seasonal effect was seen the summer/winter differences were not significant ($p=0.125$, 88% CI[7.4 -46.7]). A significant increasing trend was seen in the ANKLE SURGERY time series ($p<0.001$), with no seasonal effects detected. Searches for ANKLE ORTHOSES terms were 3.8 and 2.6 more popular than ANKLE EXERCISE and ANKLE SURGERY over the time period.

Discussion

We carried out a time series analysis of internet search terms relating to foot and ankle pain and found a number of notable trends and seasonal effects in the data. To our knowledge, this is the first study to investigate online search behaviour in relation to musculoskeletal pain. Our results suggest that online search volumes for foot and ankle pain related terms increase in the summer months. There could be a number of reasons for this seasonal pattern of increases. Perhaps the most plausible is that the summer months is when people tend to be most active, and take part in sporting and leisure activities where overuse and acute injuries are more likely to occur [5],[6]. Although evidence is lacking, it is commonly thought that cold and damp weather can cause joints to become stiffer and more painful in chronic conditions [4], however we did not see any reflection of this in our analysis. It is possible that the confounding influence of factors such as activity may hide such effects, and a more specific analysis may be required to test this hypothesis. Interestingly, the seasonal effects seen for general pain terms were most strongly reflected in the FOOT SURGERY terms, and to a lesser extent ANKLE ORTHOSES terms. The remaining treatment terms failed to show significant annual seasonal effects. Whether this is a reflection that a significant percentage of the searches relate to transient foot or ankle pain that resolves quickly and naturally without needing an intervention or further healthcare attention requires further investigation.

Increasing individual trends should be interpreted with caution as these likely relate to the increasing number of internet users seen over the study period. However, of note is the finding that the search frequency for the INSOLE terms roughly doubled over the time period studied, while FOOT ORTHOTIC terms decreased in relative popularity. This may suggest that, while for some clinicians and researchers these refer to distinct types of devices, “insole” is the preferred umbrella-term for all of these types of in-shoe interventions within the general population.

We attempted to use data from Australia to validate findings from the northern hemisphere, where data from a much larger sample population was being studied. However the time series available for many of the terms was too short to perform any meaningful analysis. Despite this, in all but one case (ANKLE SPRAIN) the Australian data tended to support the findings from the northern hemisphere countries, giving us some confidence in the robustness of these findings.

To as great an extent possible we used lay terms in our search strings. Arguably, an exception to this would be PLANTAR FACIITIS, and this may largely explain the 20-fold difference between PLANTAR FACIITIS and ANKLE SPRAIN. It is likely that a non-trivial proportion of searches for HEEL PAIN terms would relate to a condition that would be clinically defined as plantar faciitis. Epidemiological data in the literature for these conditions suggest that the relative incidence of plantar faciitis is around 5 times greater than ankle sprain [14, 15], although the more chronic nature of plantar faciitis may skew this somewhat. Additionally, search strings were limited to English language only, and while Google Trends does account for common misspellings, we did not attempt to capture the full range of potential misspellings that could occur. Google Trends is based on data from a subset of the population, those who use the Google search engine. A 2014 survey on internet use found that in the United States, 56% of those over 65 years of age used the internet, compared to 97% of those aged between 18 and 27 [16]. This potentially biases our sample in age groups who may have a higher prevalence of foot and ankle pain [1]. It should also be noted that the Google Trends tool is also updated regularly. An example of this occurred in 2011, when regional coverage was improved, and this may explain the limited data from Australia up until this point. We have included all the data used for our analyses in the supplementary material to this paper to account for any further changes to the tool.

For healthcare providers, these time series data relating to foot and ankle pain and its treatments may correlate with the number of cases of foot and ankle problems attending clinics, and by looking at the

seasonal and regional trends seen here, could potentially allow more efficient allocation of resources to suit predicted volumes of patients during different months. Clinicians and researchers should be aware of the strong interest in medical information relating to foot and ankle pain on the internet. It should also be noted that the quality of the information relating to medical problems that is available online is highly variable. It has been shown that medical information found online even on reputable websites is often poorly supported by the evidence, emphasising the need for greater efforts in providing evidence based sources [17].

Future work will look to expand this approach to other sites of musculoskeletal pain, and to access additional sources of data to determine how these findings reflect clinical demands at a condition-specific level.

Details of contributors

ST devised the study, wrote the statistical analysis plan, cleaned and analysed the data, and drafted and revised the paper. He is the guarantor. JW monitored data collection, analysed the data, and drafted and revised the paper.

Competing interest declaration

ST and JW have no competing interests that may be relevant to the submitted work.

Funding

ST is funded through the People Programme (Marie Skłodowska-Curie Actions) of the European Union's Seventh Framework Programme (FP7 2007-2013) under REA Grant Agreement No. PIOF-GA-2012-329133. The funders had no input into the design, analysis, or decision to publish.

References

1. Thomas MJ, Roddy E, Zhang W, Menz HB, Hannan MT, Peat GM: **The population prevalence of foot and ankle pain in middle and old age: a systematic review.** *Pain* 2011, **152**:2870–80.

2. Paul L, Rafferty D, Marshall-McKenna R, Gill JMR, McInnes I, Porter D, Woodburn J: **Oxygen cost of walking, physical activity, and sedentary behaviours in rheumatoid arthritis.** *Scand J Rheumatol* 2014, **43**:28–34.

3. Rupp I, Boshuizen HC, Dinant HJ, Jacobi CE, van den Bos GAM: **Disability and health-related quality of life among patients with rheumatoid arthritis: association with radiographic joint damage, disease activity, pain, and depressive symptoms.** *Scand J Rheumatol* , **35**:175–81.

4. Hawley DJ, Wolfe F, Lue FA, Moldofsky H: **Seasonal symptom severity in patients with rheumatic diseases: a study of 1,424 patients.** *J Rheumatol* 2001, **28**:1900–9.

5. O'Connell SE, Griffiths PL, Clemes SA: **Seasonal variation in physical activity, sedentary behaviour and sleep in a sample of UK adults.** *Ann Hum Biol* , **41**:1–8.

6. Tucker P, Gilliland J: **The effect of season and weather on physical activity: a systematic review.** *Public Health* 2007, **121**:909–22.

7. Bratucu R, Gheorghe I, Purcarea R, Gheorghe C, Popa Velea O, Purcarea V: **Cause and effect: the linkage between the health information seeking behavior and the online environment- a review.** *J Med Life* 2014, **7**:310–316.

8. Powell J, Inglis N, Ronnie J, Large S: **The characteristics and motivations of online health information seekers: cross-sectional survey and qualitative interview study.** *J Med Internet Res* 2011, **13**:e20.

9. Nuti S V, Wayda B, Ranasinghe I, Wang S, Dreyer RP, Chen SI, Murugiah K: **The use of google trends in health care research: a systematic review.** *PLoS One* 2014, **9**:e109583.

10. Carneiro HA, Mylonakis E: **Google trends: a web-based tool for real-time surveillance of disease outbreaks.** *Clin Infect Dis* 2009, **49**:1557–64.

11. R Development Core Team: **R: A Language and Environment for Statistical Computing**. 2014.

12. Wickham H: *ggplot2: Elegant Graphics for Data Analysis. Springer New York*. New York: Springer; 2009.

13. **Percentage of individuals using the internet** [http://www.itu.int/en/ITU-D/Statistics/Pages/stat/default.aspx] Accessed: 2014/11/08

14. Scher DL, Belmont PJ, Bear R, Mountcastle SB, Orr JD, Owens BD: **The incidence of plantar fasciitis in the United States military.** *J Bone Joint Surg Am* 2009, **91**:2867–72.

15. Waterman BR, Owens BD, Davey S, Zacchilli MA, Belmont PJ: **The epidemiology of ankle sprains in the United States.** *J Bone Joint Surg Am* 2010, **92**:2279–84.

16. **Pew Research Center Internet Project Survey** [http://www.pewinternet.org/data-trend/internet-use/latest-stats/] Accessed 2015/03/05

17. Moshirfar A, Campbell JT, Khasraghi FA, Wenz JF: **Evaluating the quality of Internet-derived information on plantar fasciitis.** *Clin Orthop Relat Res* 2004:60–3.

Table 1 – Search strategies

Group	Search reference	Search string
Pain	FOOT PAIN	"foot pain" + "painful foot" + "feet pain" + "painful feet" + "sore foot" + "sore feet"
	ANKLE PAIN	"ankle pain" + "painful ankle" + "painful ankles" + "sore ankle" + "sore ankles"
	HEEL PAIN	"heel pain" + "painful heel" + "painful heels"
Specific conditions	PLANTAR FACIITIS	"plantar faciitis"
	ANKLE SPRAIN	"ankle sprain" + "ankle sprains" + "sprained ankle" + "sprained ankles"
Foot treatments	FOOT ORTHOTIC	"foot orthotic" + "foot orthotics" + "foot orthosis" +"foot orthoses"
	INSOLE	insole + insoles
	FOOT SURGERY	"foot surgery" + "surgery on feet" + "surgical operation foot" + "surgical operations foot" + "surgical operations feet"
Ankle treatments	ANKLE ORTHOSES	"ankle orthosis" + "ankle orthoses" + "ankle orthotic" + "ankle orthotics" + "ankle foot orthosis" + "ankle foot orthoses" + "ankle foot orthotic" + "ankle foot orthotics" + "ankle splint" + "ankle splints" + "ankle brace" + "ankle braces"
	ANKLE EXERCISES	"ankle exercise" + "ankle exercises" + "exercises for ankle" + "exercises for ankles" + "ankle strengthening" + "strengthening ankle" + "strengthening ankles" + "strengthen ankle" + "strengthen ankles"
	ANKLE STRETCHES	"ankle stretches" + "ankle stretch" + "stretches for ankle" + "stretches for ankles" + "stretch ankle" + "stretch ankles" + "stretching ankle" + "stretching ankles" + "ankle stretching"
	ANKLE SURGERY	"ankle surgery" + "surgery on ankle" + "surgery on ankles" + "ankle operation" + "ankle replacement" + "surgical operation ankle" + "surgical operation ankles"

Note: In Google Trends the + symbol represents OR in Boolean notation. Additionally, enclosing more than one word in quotation marks ensures that the exact phrase is searched for and not just the presence of those words in any order within a larger phrase.

Table 2: Time series analysis of search terms

Search reference	Location	Mann Kendall trend test	TBATS (seasonality present?)	Summer median	Winter median	Wilcoxon signed rank test (winter/summer)
FOOT PAIN	NH	$p<0.001$, tau=0.472	Yes	103.5	76.47	$p=0.004$ 95% CI[22.2-32.1]
	AUS	$p<0.001$, tau=0.221	Yes	101.90	84.23	$p=0.25$ 88% CI[-3.6-41.9]
ANKLE PAIN	NH	$p<0.001$, tau=0.531	Yes	94.02	71.88	$p=0.0078$ 95% CI[20.9-35.5]
	AUS	$p=0.9$, tau=-0.007	No	100.20	93.61	-
HEEL PAIN	NH	$p<0.001$, tau=0.278	Yes	112.80	71.49	$p=0.004$ 95% CI[29.1-45.6]
	AUS	0.005, tau=0.144	Yes	104.70	83.85	$p=0.25$ 75% CI[15.9-21.4]
PLANTAR FACIITIS	NH	$p<0.001$, tau=0.598	Yes	105.30	80.98	$p=0.004$ 95% CI[21.5-37.4]
	AUS	$p<0.001$, tau=0.361	Yes	91.63	85.13	$p=0.063$ 95% CI[5.2 - 13.6]
ANKLE SPRAIN	NH	$p<0.001$, tau=0.535	Yes	98.95	74.24	$p=0.004$, $W=45$ 95% CI[14.5-33.7]
	AUS	$p=0.027$, tau=0.096	Yes	91.55	99.76	$p=0.125$ 88% CI[7.6-18.7]
FOOT ORTHOTIC	NH	$p=0.001$, tau=-0.157	No	98.87	96.34	-
	AUS	-	-			-
INSOLE	NH	$p<0.001$, tau=0.628	No	84.26	93.48	-
	AUS	$p<0.001$, tau=0.276	No	95.62	92.73	-
FOOT SURGERY	NH	$p=0.415$, tau=-0.028	Yes	105.30	89.58	$p=0.031$ 95% CI[3.5-20.9]
	AUS	-	-			-
ANKLE ORTHOSES	NH	$p<0.001$, tau=0.354	Yes	97.64	90.07	$p=0.004$ 95% CI[7.6-25.2]
	AUS	-	-			-
ANKLE EXERCISES	NH	$p=0.067$, tau=0.078	Yes	99.82	76.32	$p=0.125$ 88% CI[7.4 -46.7]
	AUS	-	-			-
ANKLE SURGERY	NH	$p<0.001$, tau=0.159	No	103.20	88.27	-
	AUS	-	-			-

Summer and winter medians are unitless. NH: northern hemisphere; AUS: Australia

Figure legends

Figure 1 – Relative search volume of foot and ankle pain related terms. Shaded lines are weekly data; bold lines are a 5 week moving average. Note the reversal of minima/maxima in Australia (southern hemisphere). NH: northern hemisphere; AUS: Australia

Figure 2 – Local regression based decomposition of time series for pain terms. From left to right, these present results for FOOT PAIN, ANKLE PAIN, and HEEL PAIN. The top row represents northern hemisphere data, and the bottom data for Australia. Periodograms are included as the lowest subplot for each term/region.

Figure 3 – Relative search volume of foot and ankle pain related terms. Shaded lines are weekly data; bold lines are a 5 week moving average. NH: northern hemisphere; AUS: Australia

Figure 4 – Local regression based decomposition of time series for pain terms. From left to right, these present results for PLANTAR FACIITIS and ANKLE SPRAIN. The top row represents northern hemisphere data, and the bottom data for Australia. Periodograms are included as the lowest subplot for each term/region.

Figure 5 – Relative search volume of foot and ankle pain related terms. Shaded lines are weekly data; bold lines are a 5 week moving average. NH: northern hemisphere; AUS: Australia

Figure 6 – Local regression based decomposition of time series for pain terms. From left to right, these present results for FOOT ORTHOTIC, INSOLE, and FOOT SURGERY. The top row represents northern hemisphere data, and, where available, the bottom data for Australia. Periodograms are included as the lowest subplot for each term/region.

Figure 7 – Relative search volume of foot and ankle pain related terms. Shaded lines are weekly data; bold lines are a 5 week moving average.

Figure 8 – Local regression based decomposition of time series for pain terms. From left to right, these present results for ANKLE ORTHOSES, ANKLE EXERCISES, and ANKLE SURGERY in the northern hemisphere. Periodograms are included as the lowest subplot for each term.

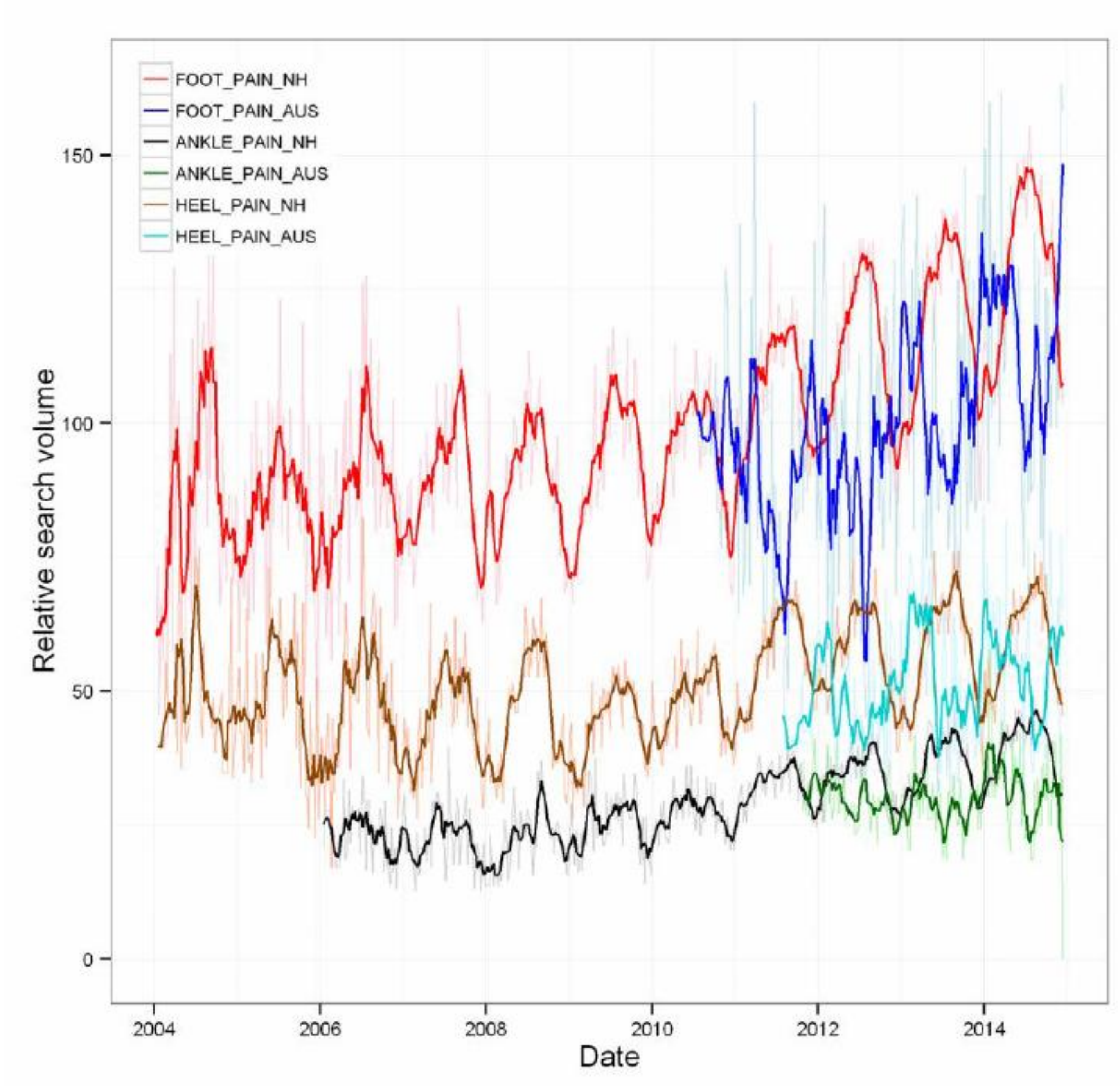
FOOT_PAIN_NH
FOOT_PAIN_AUS
ANKLE_PAIN_NH
ANKLE_PAIN_AUS
HEEL_PAIN_NH
HEEL_PAIN_AUS
150
100
50
0
Relative search volume
2004
2006
2008
2010
2012
2014
Date

Figure 1

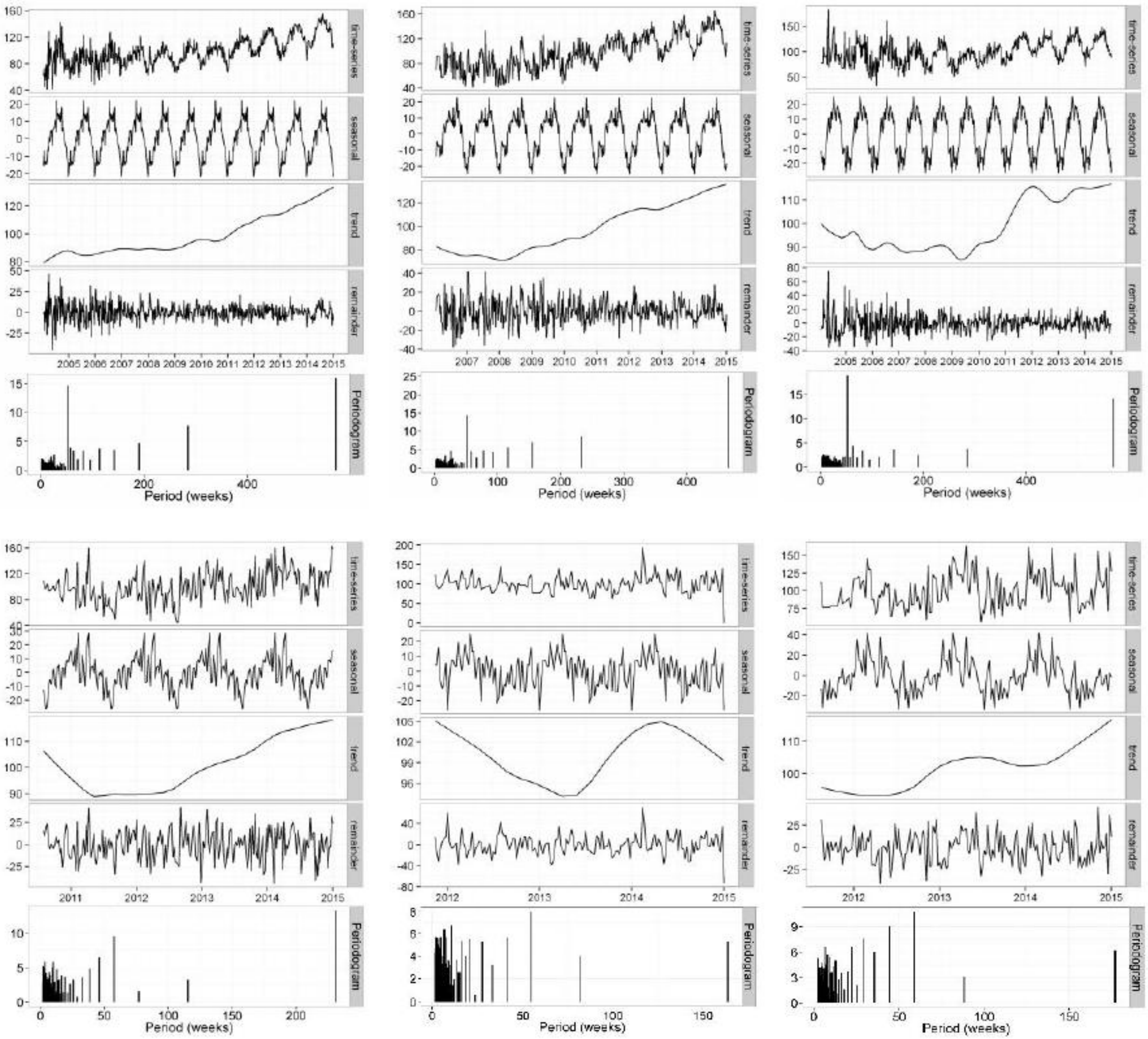

time-series
seasonal
trend
remainder
2005 2006 2007 2008 2009 2010 2011 2012 2013 2014 2015
Periodogram
Period (weeks)
time-series
seasonal
trend
remainder
2007 2008 2009 2010 2011 2012 2013 2014 2015
Periodogram
Period (weeks)
time-series
seasonal
trend
remainder
2005 2006 2007 2008 2009 2010 2011 2012 2013 2014 2015
Periodogram
Period (weeks)
time-series
seasonal
trend
remainder
2011 2012 2013 2014 2015
Periodogram
Period (weeks)
time-series
seasonal
trend
remainder
2012 2013 2014 2015
Periodogram
Period (weeks)
time-series
seasonal
trend
remainder
2012 2013 2014 2015
Periodogram
Period (weeks)

Figure 2

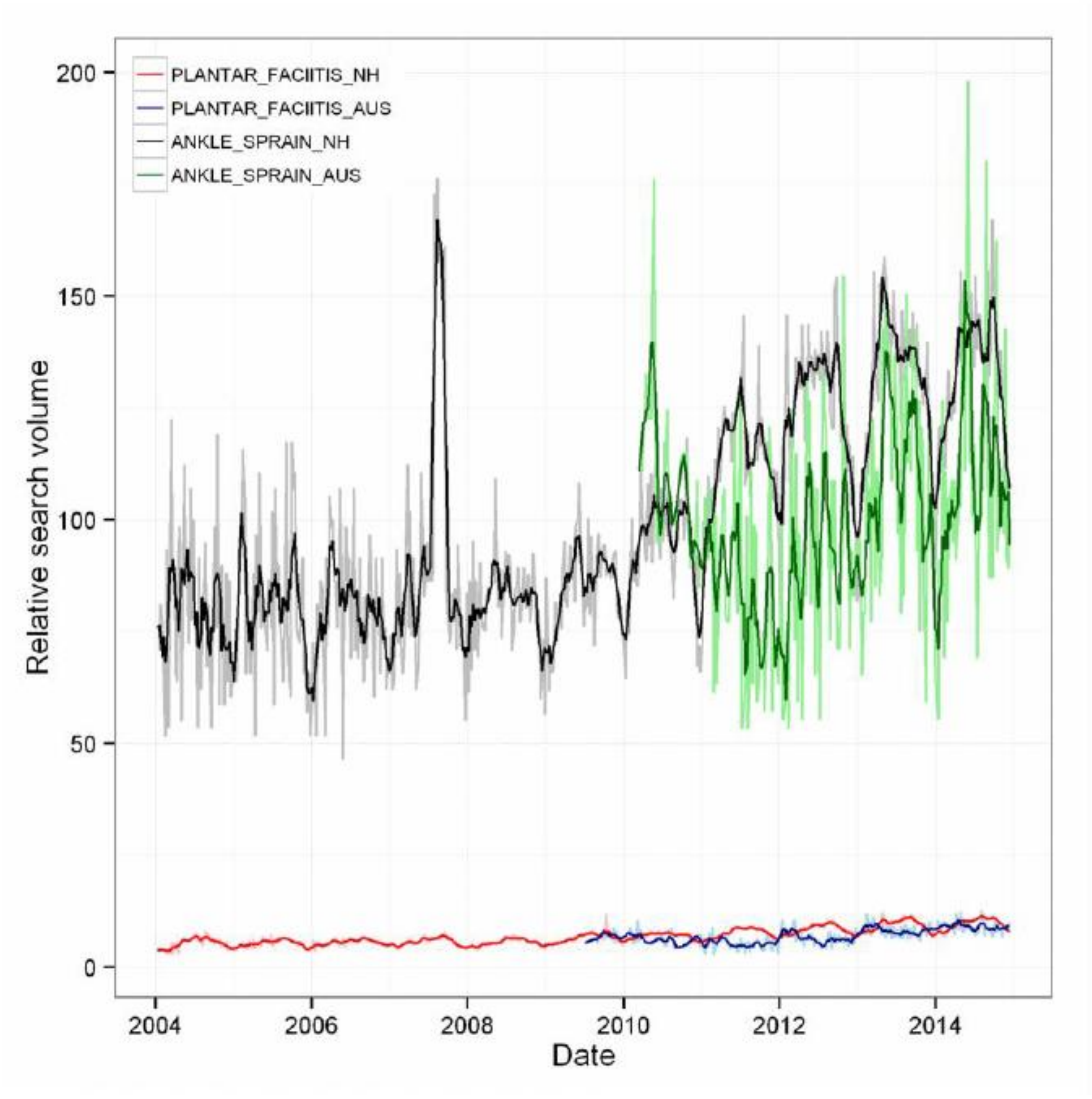
PLANTAR_FACIITIS_NH
PLANTAR_FACIITIS_AUS
ANKLE_SPRAIN_NH
ANKLE_SPRAIN_AUS
Relative search volume
200
150
100
50
0
2004
2006
2008
2010
2012
2014
Date

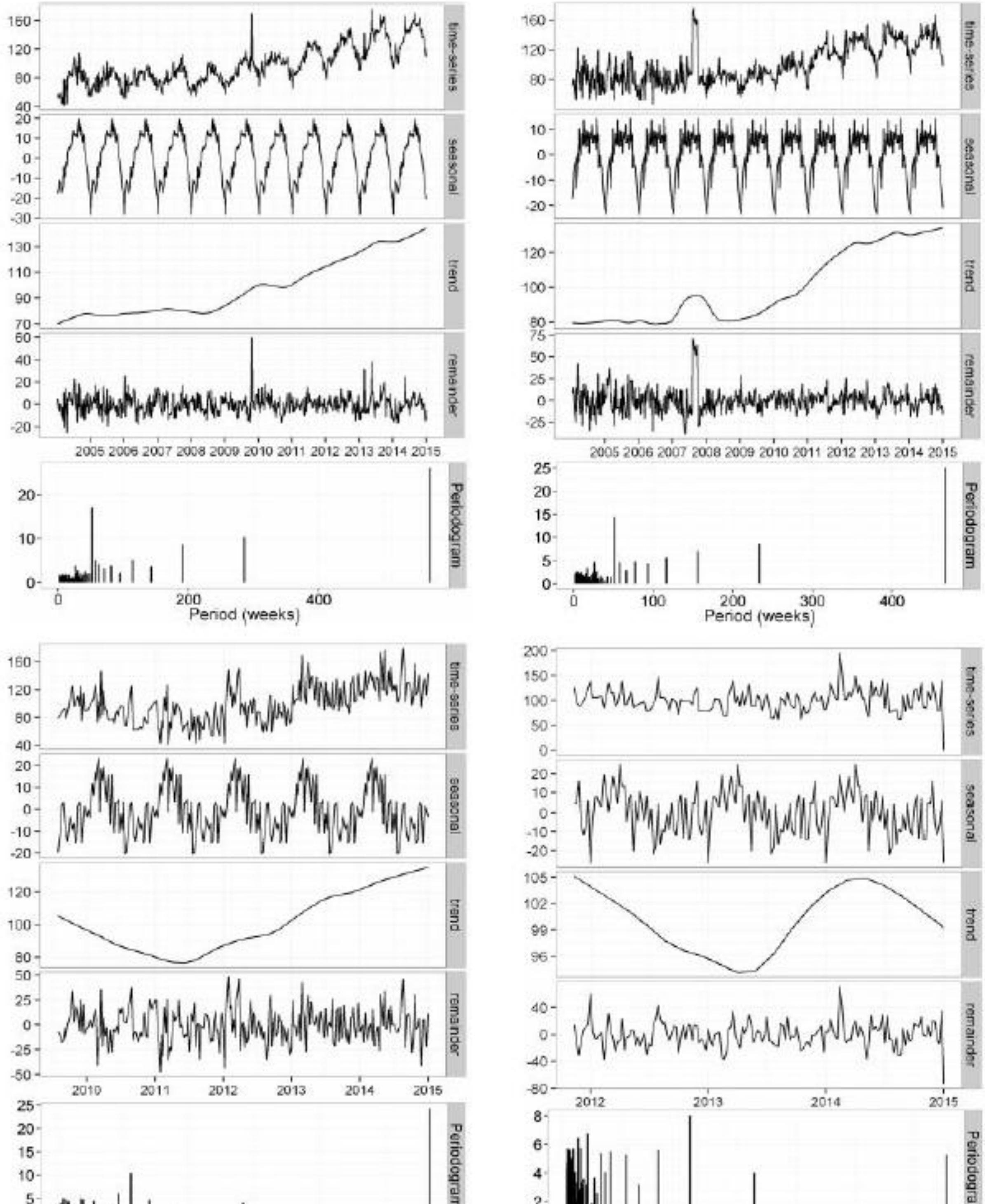
time-series
seasonal
trend
remainder
2005 2006 2007 2008 2009 2010 2011 2012 2013 2014 2015
Periodogram
Period (weeks)
time-series
seasonal
trend
remainder
2005 2006 2007 2008 2009 2010 2011 2012 2013 2014 2015
Periodogram
Period (weeks)
time-series
seasonal
trend
remainder
2010 2011 2012 2013 2014 2015
Periodogram
time-series
seasonal
trend
remainder
2012 2013 2014 2015

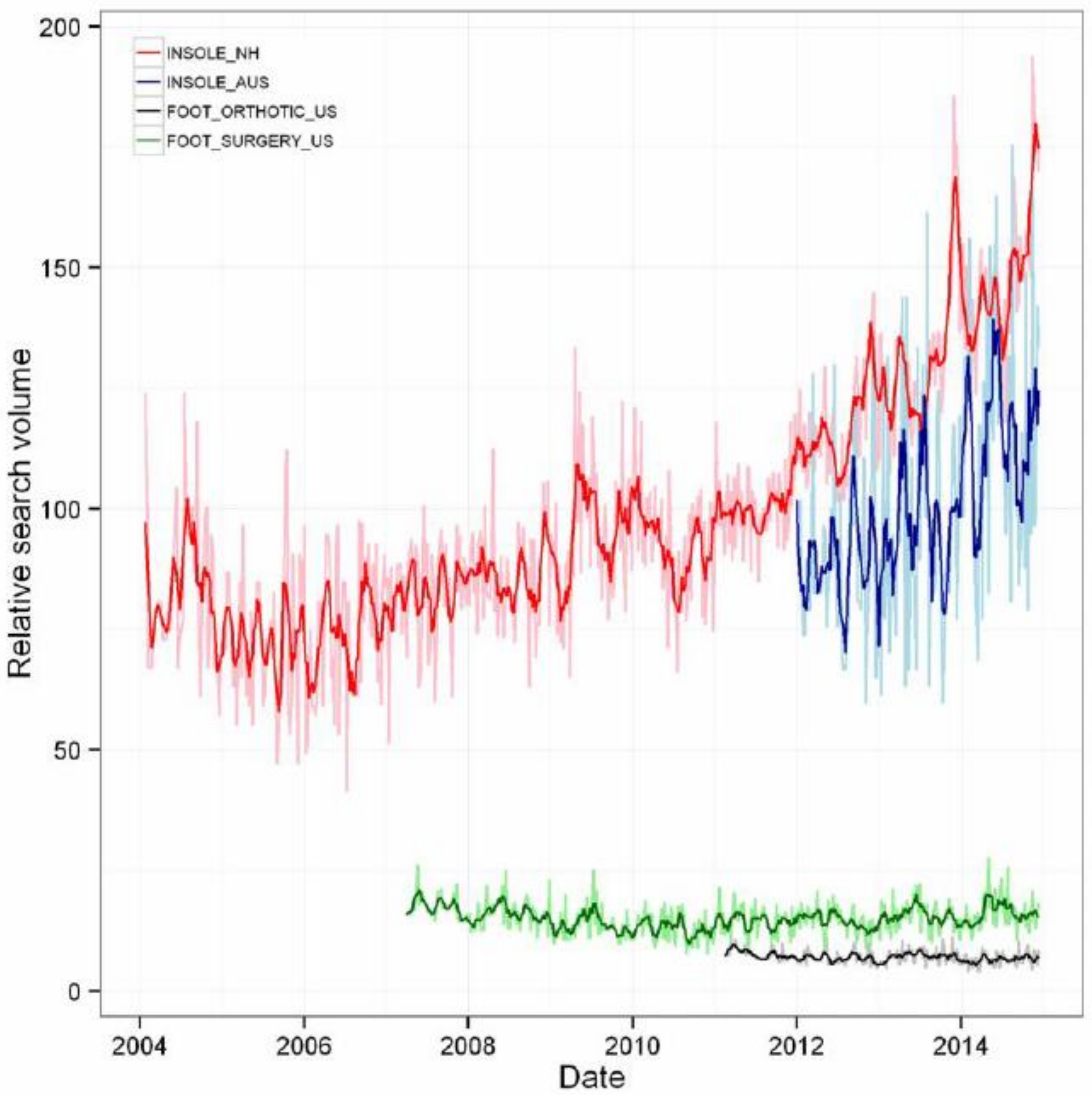

INSOLE_NH
INSOLE_AUS
FOOT_ORTHOTIC_US
FOOT_SURGERY_US
Relative search volume
200
150
100
50
0
2004
2006
2008
2010
2012
2014
Date

Figure 5

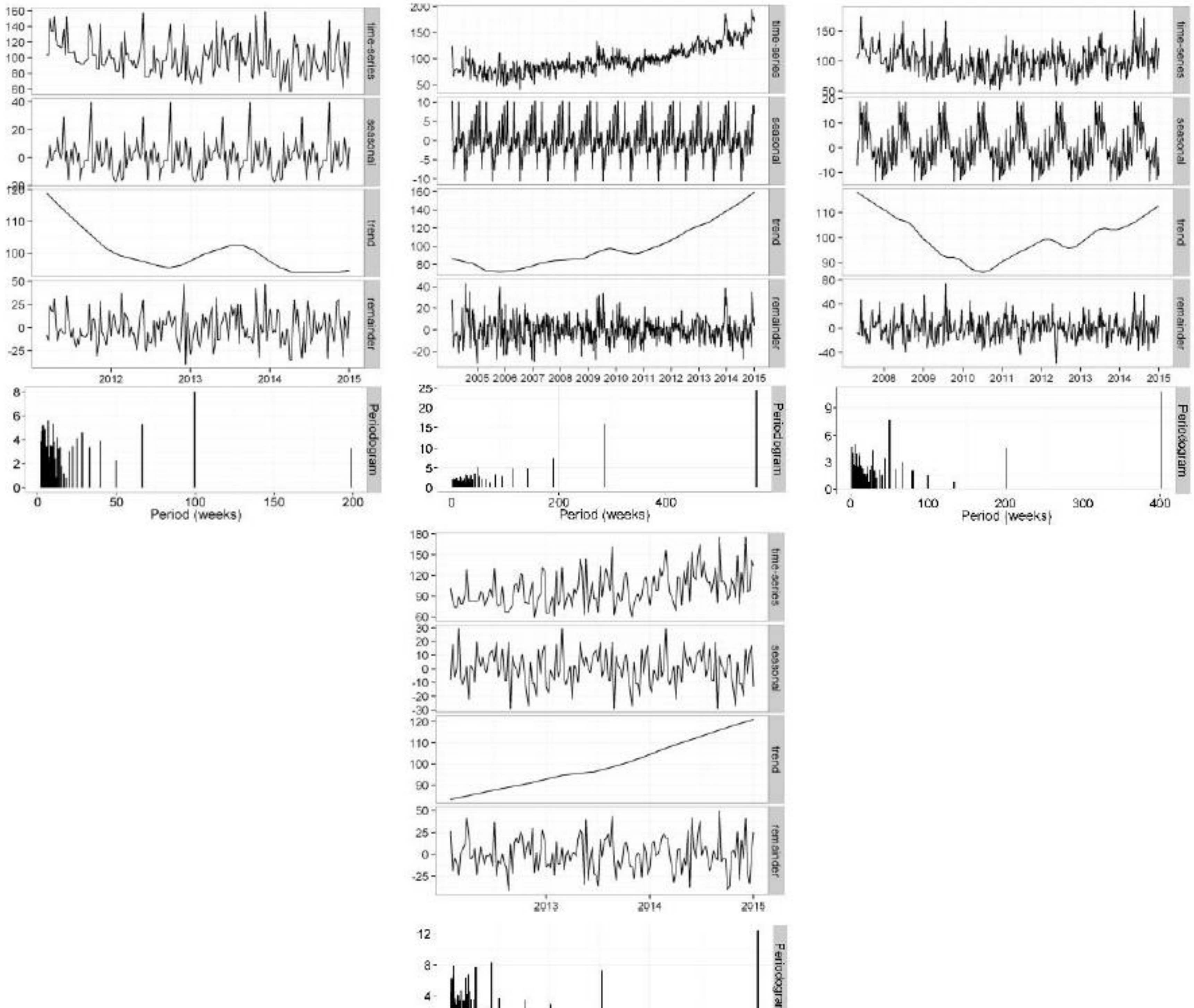
time-series
seasonal
trend
remainder
Periodogram
Period (weeks)

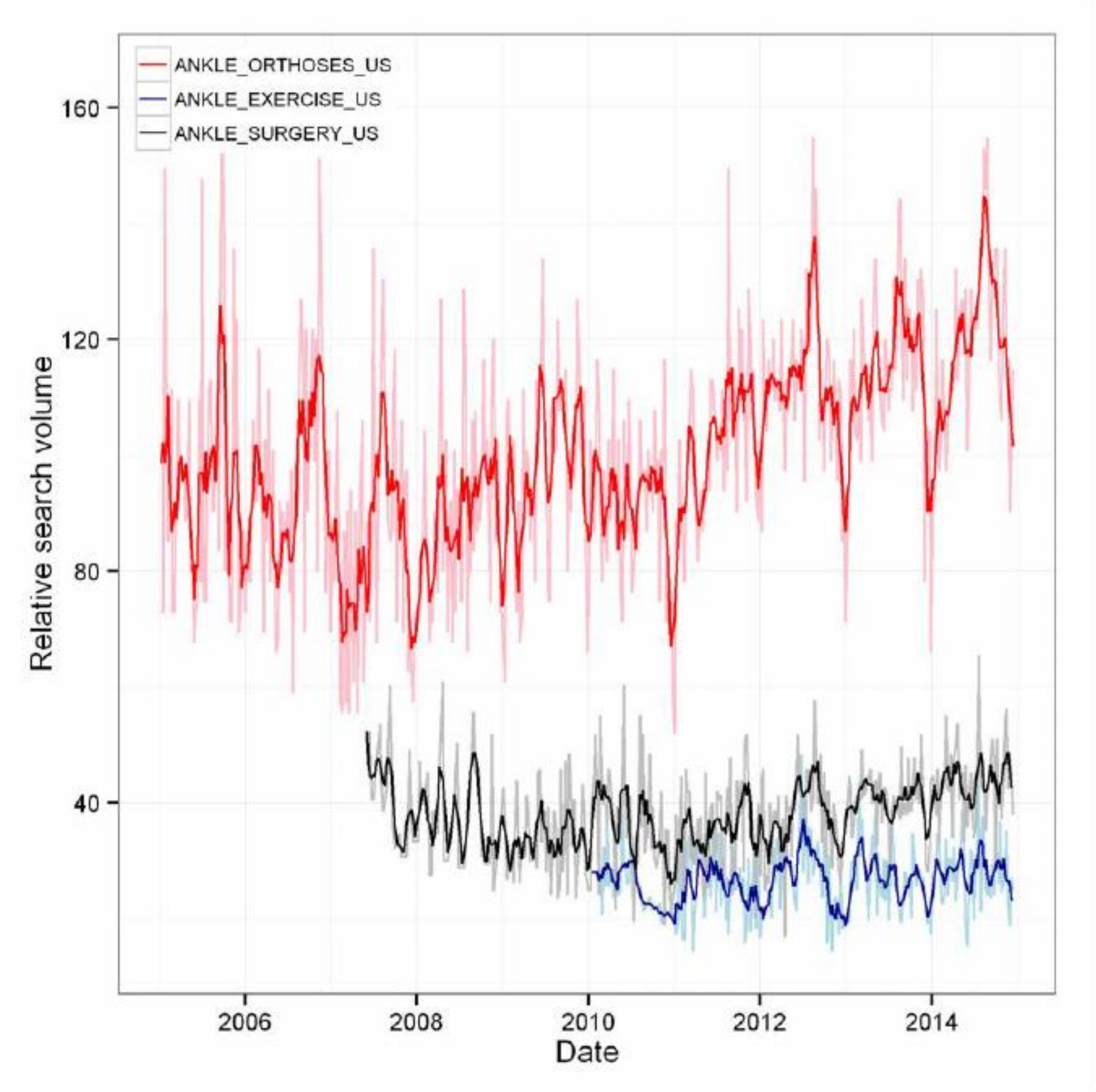
ANKLE_ORTHOSES_US
ANKLE_EXERCISE_US
ANKLE_SURGERY_US
160
120
80
40
Relative search volume
2006
2008
2010
2012
2014
Date

Figure 7

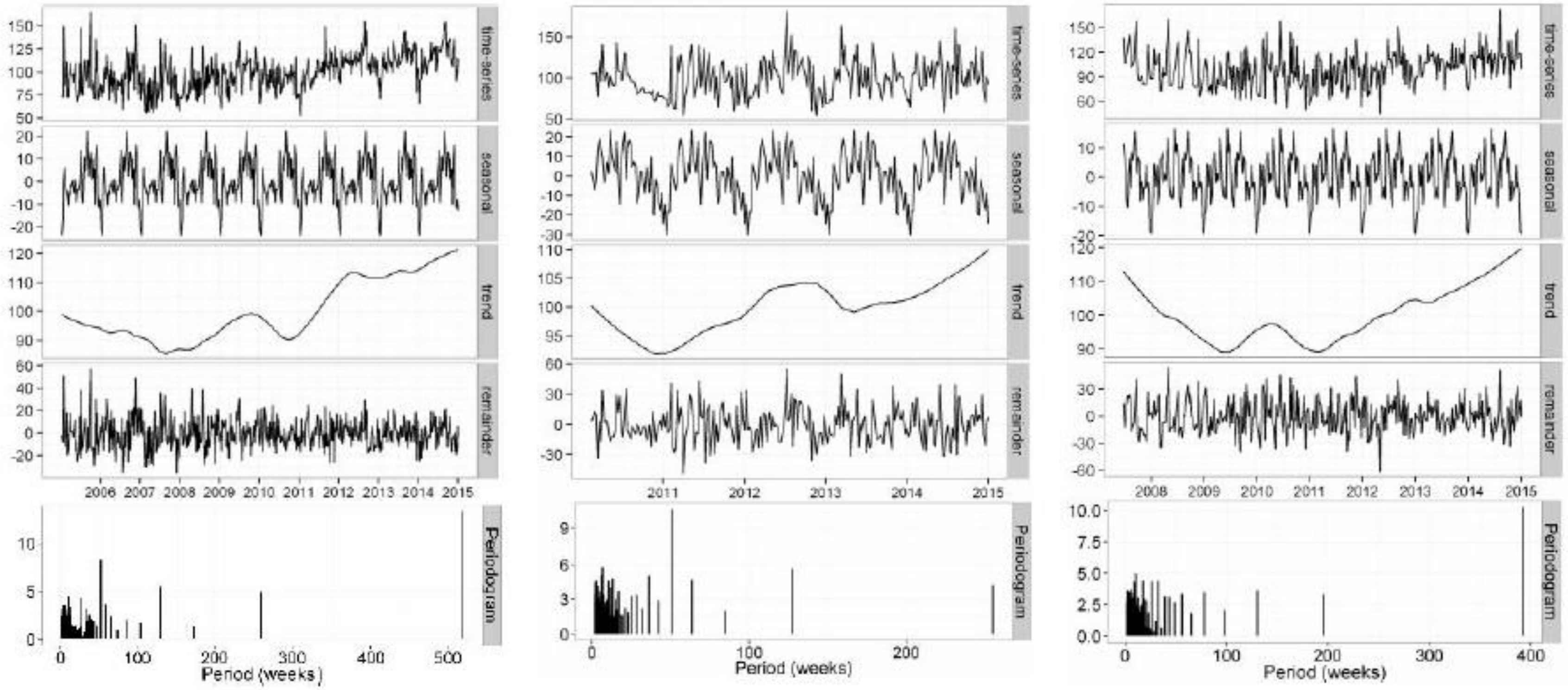
time-series
seasonal
trend
remainder
Periodogram
Period (weeks)

Figure 8
